# Expression signature of lncRNAs and their potential roles in cardiac fibrosis of post-infarct mice

**DOI:** 10.1042/BSR20150278

**Published:** 2016-06-03

**Authors:** Xuefeng Qu, Xiaotong Song, Wei Yuan, You Shu, Yuying Wang, Xuyun Zhao, Ming Gao, Renzhong Lu, Shenjian Luo, Wei Zhao, Yue Zhang, Lihua Sun, Yanjie Lu

**Affiliations:** * Department of Pharmacology (State-Province Key Laboratories of Biomedicine-Pharmaceutics of China, Key Laboratory of Cardiovascular Research, Ministry of Education), College of Pharmacy, Harbin Medical University, Harbin, Heilongjiang 150081, P.R. China; † Northern Translational Medicine Research and Cooperation Center, Heilongjiang Academy of Medical Sciences, Harbin Medical University, Harbin, Heilongjiang 150081, P.R. China

**Keywords:** cardiac fibrosis, expression, gene, long non-coding RNA (lncRNA), myocardial infarction

## Abstract

The present study aimed to investigate whether long non-coding RNAs (lncRNAs) are involved in cardiac fibrogenesis induced by myocardial infarction (MI). The differentially expressed lncRNAs and mRNAs in peri-infarct region of mice 4 weeks after MI were selected for bioinformatic analysis including gene ontology (GO) enrichment, pathway and network analysis. Left ventricular tissue levels of lncRNAs and mRNAs were compared between MI and sham control mice, using a false discovery rate (FDR) of <5%. Out of 55000 lncRNAs detected, 263 were significantly up-regulated and 282 down-regulated. Out of 23000 mRNAs detected, 142 were significantly up-regulated and 67 down-regulated. Among the differentially expressed lncRNAs, 53 were up-regulated by ≥2.0-fold change and 37 down-regulated by ≤0.5-fold change. Nine up-regulated and five down-regulated lncRNAs were randomly selected for quantitative real-time PCR (qRT-PCR) verification. GO and pathway analyses revealed 173 correlated lncRNA–mRNA pairs for 57 differentially expressed lncRNAs and 20 differentially expressed genes which are related to the development of cardiac fibrosis. We identified TGF-β_3_ as the top-ranked gene, a critical component of the transforming growth factor-β (TGF-β) and mitogen activated protein kinase (MAPK) signalling pathways in cardiac fibrosis. NONMMUT022554 was identified as the top-ranked lncRNA, positively correlated with six up-regulated genes, which are involved in the extracellular matrix (ECM)–receptor interactions and the phosphoinositid-3 kinase/protein kinase B (PI3K-Akt) signalling pathway. Our study has identified the expression signature of lncRNAs in cardiac fibrosis induced by MI and unravelled the possible involvement of the deregulated lncRNAs in cardiac fibrosis and the associated pathological processes.

## Abstract (precis)

Myocardial fibrotic tissue from mouse infarcted heart displayed the expression signature of long non-coding RNAs (lncRNAs) and mRNAs. Fifty-seven differentially expressed lncRNAs and 20 differentially expressed mRNAs were found to be related to 8 signalling pathways involved in the development of cardiac fibrosis.

## INTRODUCTION

Cardiac fibrosis is an important event of the cardiac remodelling process with increased activity of cardiac fibroblasts and production of extracellular matrix (ECM) within the myocardium, which can lead to arrhythmias and heart failure [1]. Myocardial infarction (MI) is characterized by cardiac remodelling involving apoptosis, hypertrophy and fibrosis. Interstitial fibrosis is a major pathological alteration of post-infarct remodelling. The underlying mechanisms of cardiac fibrosis include activation of transforming growth factor-β (TGF-β) [2], endothelin-1 [3], angiotensin II (AngII) [4] and platelet-derived growth factor [5], and excess production and deposition of ECM proteins (including collagen I, collagen III, MMP2 and MMP9) in the myocardium [6]. Many cellular factors have been identified as important regulators of cardiac fibrosis, including protein and non-protein molecules, such as miRNAs [7–9] and long non-coding RNAs (lncRNAs) [10,11]. However, the precise molecular mechanisms of cardiac fibrosis remain to be fully understood.

LncRNAs are defined as transcripts larger than 200 nucleotides without protein coding ability [12,13]. A large number of evidence revealed that lncRNAs participate in diverse biological processes [14–17] and play important roles in various diseases including cancer [18–22], Alzheimer's disease [23], diabetes [24], cardiac hypertrophy [25] and heart failure [26]. It has been reported that a lncRNA named cardiac hypertrophy-related factor (CHRF) plays an important role in cardiac hypertrophy through its sponge-like action on *miR-489* [25]. Wang et al. [27] demonstrated that another lncRNA, cardiac apoptosis-related lncRNA (CARL), regulates apoptosis by targeting *miR-539* and PHB2 in mice with MI. More recently, the same group further demonstrated that autophagy promoting factor (APF) regulates autophagic cell death by targeting *miR-188-3p* and ATG7 [28]. To date, there is only one published study relating lncRNAs to cardiac fibrosis showing the altered expression of lncRNAs in AngII-treated cardiac fibroblasts [29]. However, research on roles of lncRNAs in cardiac fibrosis is still lacking.

In the present study, we identified differentially expressed lncRNAs in a mouse model of cardiac fibrosis induced by MI. We also performed gene ontology (GO) and pathway analyses for functional annotation of the deregulated lncRNAs and established the lncRNA–mRNA co-expression network in a mouse model of cardiac fibrosis induced by MI.

## MATERIALS AND METHODS

### Mouse model of myocardial infarction

Male C57BL/6 mice were purchased from the Second Affiliated Hospital of Harbin Medical University and experimental protocols were in accordance with and approved by the Institutional Animal Care and Ethics Committee of the Harbin Medical University, P.R. China. MI was induced by coronary ligation [7]. Briefly, animals were anesthetized using avertin (160 mg/kg, i.p.; Sigma–Aldrich). A left-sided thoracotomy was performed to expose the heart, and the left descending coronary artery was ligated using 7/0 silk thread at a level approximately 2 mm below the edge of the left auricle. Ischemia was confirmed by significant S–T segment elevation in electrocardiogram. The sham-operation group underwent identical protocols without ligating the coronary artery. After surgery mice were monitored daily for signs of infection and state of health and activity for 4 weeks.

### RNA extraction

For microarray analysis, peri-infarct region (1–2 mm area between the infarct region and normal tissue) and corresponding regions of sham-operated hearts were collected 4 weeks after MI. Total RNA was extracted by using Trizol reagent (Invitrogen) according to the manufacturer's instructions. In brief, RNA sample was sit for 3 min at room temperature to allow complete dissociation of the nucleoprotein complex, following addition of 0.2 ml chloroform/1 ml of Trizol reagent. The sample was centrifuged at 16 500 ***g*** at 4°C for 15 min. The aqueous phase was removed and 0.5 ml of propan-2-ol was added to the tube, followed by incubation at room temperature for 10 min. The sample was again centrifuged at 16 500 ***g*** at 4°C for 10 min. Finally, the pellet was washed with 1 ml of 75% ethanol.

### Echocardiographic measurements

We measured left ventricular function in mice 4 weeks after MI using transthoracic echocardiography performed by an ultrasound machine Vevo2100 high-resolution imaging system (Visual Sonics) equipped with a 10-MHz phased-array transducer. The M-mode tracings were recorded to measure left ventricular systolic diameter (LVSd), left ventricular diastolic diameter (LVDd), left ventricular ejection fraction (EF) and fractional shortening (FS). All measurements and calculations were performed on three continuous beats.

### Masson's trichrome staining

Masson's trichrome staining was performed to evaluate collagen deposition. Mice were anaesthetized using avertin and the hearts were collected, fixed in 4% paraformalin, embedded with paraffin and then cut into 5 μm-thick cross sections along the centre of the fibrotic scar. After 24 h the sections were stained by Masson's trichrome and the staining was analysed with image analysis software (Image-Pro Plus 4.0, Media Cybernetic). We used a bright-field microscope (IX71 Olympus) to examine the collagen deposition. We used the ratio of collagen surface area to the myocardial surface area to obtain the collagen volume fraction as an index of cardiac fibrosis.

### Microarray analysis of lncRNAs and mRNAs

Total RNA was extracted from left ventricle using Trizol (Invitrogen), and RNA cleanup including a DNase I digestion step was performed using RNeasy spin columns (Qiagen). Total RNA from each sample was quantified by the NanoDrop 1000 and RNA integrity was assessed by standard denaturing agarose gel electrophoresis. Expression profiles of lncRNAs and mRNAs were analysed with three samples from peri-infarct region of MI hearts and three samples from sham-operated hearts. For microarray analysis, GeneChip mouse transcriptome assays 1.0 from Affymetrix was used to detect the expression profiles of lncRNA and mRNA, which contains 55000 mouse lncRNA genes and 23000 mRNAs. Raw signal intensities were normalized by RMA + SKETCH method using EC 2.0, and low intensity genes were filtered. The two-sample *t*-test was used to select the differentially expressed lncRNAs and mRNAs. The *P* values were adjusted with the Benjamini and Hochberg correction procedure to account for multiple tests. The lncRNAs (and mRNAs) with false discovery rate (FDR) less than 5% were defined as differentially expressed lncRNAs (and mRNAs). Then, the lncRNAs (and mRNAs) with ≥2.0 fold change or ≤0.5 fold change over the sham-operated control samples were defined as up-regulation or down-regulation. Hierarchical clustering was performed to show the distinguishable lncRNA expression profile between MI mice and sham-operated control samples.

The microarray data have been deposited in the National Center for Biotechnology Information's Gene Expression Omnibus Database under accession number GSE76387.

### Functional enrichment analyses

The potential biological function of genes was analysed by functional enrichment using the biological process terms in GO database (http://www.geneontology.org) and pathway information in Kyoto Encyclopedia of Genes and Genomes (KEGG) database (http://www.genome.jp/kegg/pathway.html). Fisher's exact test was used to evaluate whether the GO terms or the KEGG pathways were enriched with the differentially expressed genes. And the Benjamini and Hochberg correction procedure was used to calculate the adjusted *P* values and the FDR < 5% was set as the cut-off threshold.

### Construction of the co-expression network

Differentially expressed lncRNAs and mRNAs were filtered by fold change ≥2.0 or ≤ 0.5. The Pearson correlation coefficient was used to test the expression correlation of lncRNAs and mRNAs in MI samples. Only the lncRNA–mRNA pairs with *P*<0.05 were included to construct the co-expression network. The Cytoscape software was used to present the co-expression network (http://www.cytoscape.org/).

### Quantitative real-time PCR

Quantitative real-time PCR (qRT-PCR) was performed to confirm the results of microarray analysis with a randomly selected subset of differentially expressed lncRNAs. Total RNA was extracted from peri-infarct region of MI hearts and from the sham operation group by using Trizol reagent (Invitrogen). Total RNA (1 μg) was reverse transcribed to cDNA that was subsequently used as a template in a 20 μl reaction containing 7 μl of ddH_2_O, 10 μl of 2 × SYBE Green mixture (Roche), 1 μl of 10× forward primer and 1 μl of 10× reverse primer (Invitrogen). qRT-PCR was performed with the 7500 Real-Time PCR system (Applied Biosystems) at 95°C for 10 min, followed by 40 cycles of 95°C for 15 s, 60°C for 30 s and 72°C for 30 s. β-Actin was used as an internal control for measurement of lncRNAs. The sequences of the primers used in our study are listed in Supplementary Table S1.

### Statistics

Data are expressed as mean ± S.E.M. Statistical analysis was performed using Student's *t* test. A two-tailed *P*<0.05 was taken to indicate a statistically significant difference.

## RESULTS

### Cardiac fibrosis induced by myocardial infarction

**Figure 1:**
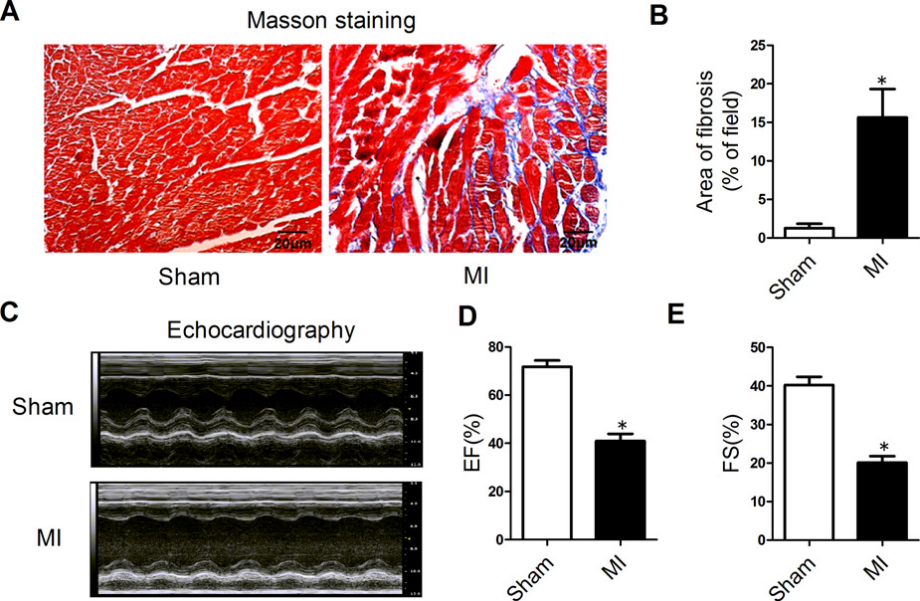
Cardiac fibrosis induced by myocardial infarction (**A**) Representative sections of heart with Masson's trichrome staining at a magnification of 400×. The fibrotic tissues were stained blue. (**B**) Collagen deposition was quantified using an automated image analyser and is expressed as the percentage of tissue area (*n*=4). (**C**) Representative M-mode echocardiography of mice 4 weeks after MI. (**D**) EF in the MI mice and sham-operated animals. (**E**) FS in the MI and sham groups (*n*=6). Data are expressed as mean ± S.E.M.; *P<0.05 compared with sham.

Masson staining of cardiac tissue sections was employed to examine cardiac fibrosis. As shown in Figure 1(A), significantly increased ECM deposition was observed in the peri-infarct region of MI hearts compared with the sham group (Figures 1A and 1B). Echocardiographic examination showed that the EF and FS were both significantly decreased in the MI mice compared with the sham mice, indicating that cardiac function had been significantly impaired (Figures 1C–1E).

### Differentially expressed lncRNAs and mRNAs

**Figure 2:**
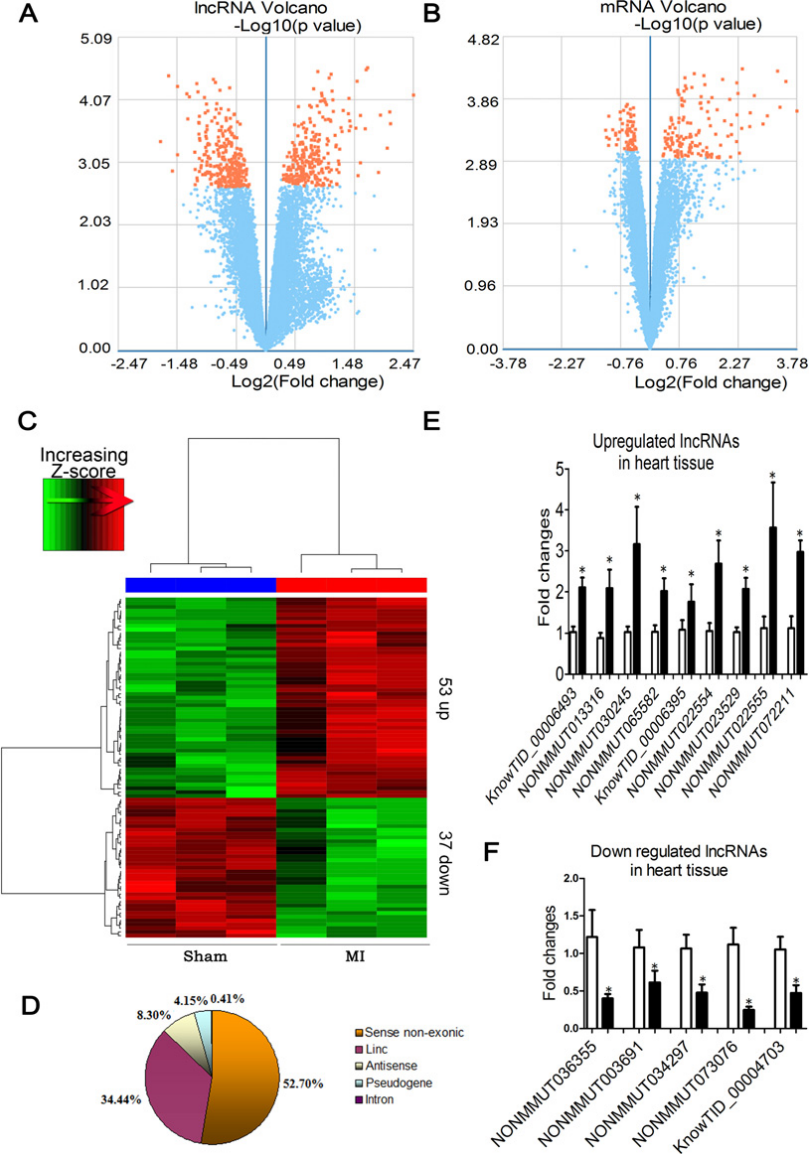
Differentially expressed lncRNAs and mRNAs between MI mice and sham control animals Volcano plot provides a convenient way to visualize the distribution of datasets of lncRNAs (**A**) and mRNAs (**B**). (**C**) The results from hierarchical clustering showing the distinct lncRNA expression profiles between MI and control. ‘Red’ indicates high relative expression and ‘green’ low relative expression. (**D**) Distribution of differentially expressed lncRNAs according to different biotypes. (**E** and **F**) Verification of the microarray results using qRT-PCR methods. Nine up-regulated and five down-regulated lncRNAs from the infracted hearts were studied using β-actin as an internal control (*n*=5–6). *P<0.05 compared with sham-operated control.

The volcano plot showed the expression alterations of all annotated genes analysed by microarray assay in peri-infarct tissue of the MI samples and the sham-operated ones. The orange dots indicate either the up- (fold change ≥ 2.0) or down-regulated (fold change ≤ 0.5) genes (Figures 2A and 2B). We found that 545 (263 up and 282 down) of 55000 detected lncRNAs and 209 (142 up and 67 down) of 23000 mRNAs showed significant differences between MI and control samples (FDR < 0.05). As shown in the heat-map (Figure 2C), among the differentially expressed lncRNAs, 53 were up-regulated with more than 2.0-fold change (Supplementary Table S2) and 37 were down-regulated with less than 0.5-fold change (Supplementary Table S3). The distribution diagram demonstrated the classification of lncRNA in the microarray (Figure 2D).

In order to verify the data obtained from our microarray analysis, quantitative real-time RT-PCR (qPCR) was applied to a randomly selected subset from the differentially expressed lncRNAs. These included nine up-regulated lncRNAs (KnowTID_00006493, NONMMUT013316, NONMMUT030245, NONMMUT065582, KnowTID_00006395, NONMMUT022554, NONMMUT023529, NONMMUT022555 and NONMMUT72211) and five down-regulated ones (NONMMUT036355, NONMMUT003691, NONMMUT034297, NONMMUT073076 and KnowTID_00004703). As shown in Figures 2(E) and 2(F), all these 14 lncRNAs were verified for their differential expression as revealed by our microarray results.

### Deregulated mRNAs that are related with cardiac fibrosis

**Figure 3:**
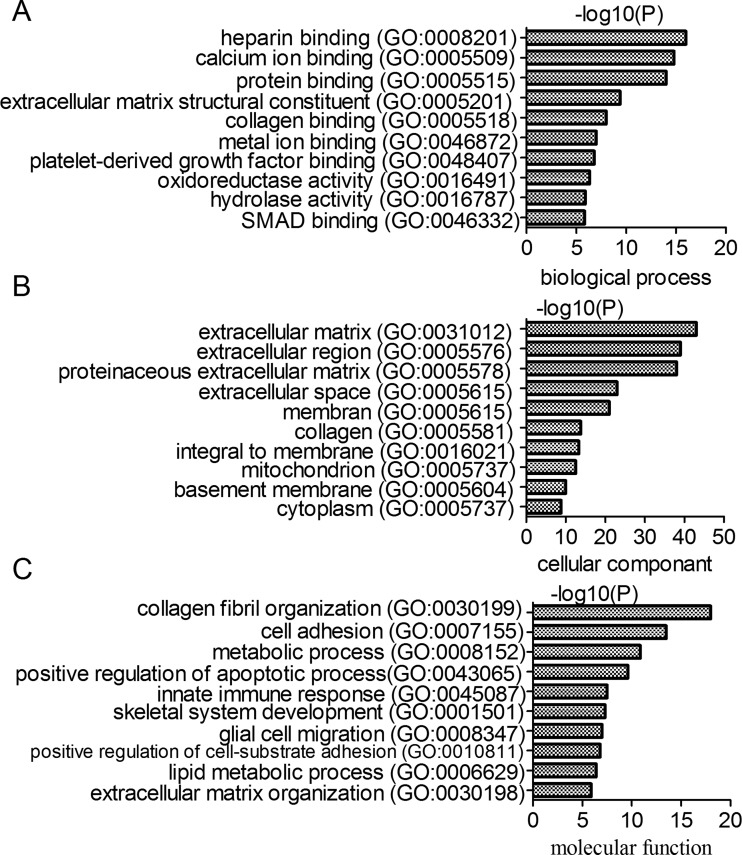
Gene ontology enrichment analysis of differentially expressed mRNAs (**A**) Biological process enrichment analysis. (**B**) Cell component enrichment analysis. (**C**) Molecular function enrichment analysis.

Enrichment analysis was performed based on the datasets of biological processes, cellular components and molecular functions from the GO database. Under the control of FDR < 5%, we found that the differentially expressed mRNAs were enriched in the ECM structural constituent and collagen binding in biological processes, ECM and extracellular region in cellular components and collagen fibril organization and cell adhesion in molecular functions (Figure 3). The differentially expressed genes were significantly involved in the cardiac fibrosis related function in GO analysis.

We next performed the pathway enrichment analysis using the KEGG database and set the cut-off as FDR < 5%. Among the significantly enriched pathways, 8 pathways annotated with 63 differentially expressed genes were closely related to the process of cardiac fibrosis (Table 1). For example, 12 differentially expressed genes (Col5a1, Itgb8, Col1a1, Col1a2, Fn1, Col5a2, Col3a1, Thbs4, Spp1, Tnc, Thbs2 and Comp) were annotated in the pathway of ECM–receptor interaction, which were mostly significantly enriched with the differentially expressed genes in the MI samples compared with the sham control ones (*P*=5.66E−16). The phosphoinositid-3 kinase/protein kinase B (PI3K-Akt) signalling pathway contained 13 differentially expressed genes (Col5a2, Col5a1, Col3a1, Comp, Thbs4, Col1a1, Thbs2, Spp1, Itgb8, Col1a2, Fn1, Tnc and Tlr4) and was significantly enriched with differentially expressed genes (*P*=8.78E−10). The TGF-β signalling pathway (annotated with Dcn, Tgfbr1 and Tgfb3), the mitogen activated protein kinase (MAPK) signalling pathway (annotated with Tgfbr1, Ecsit, Tgfb3 and Pla2g4a), the NF-kB signalling pathway (annotated with Gm21541, Gm13304, Ccl21b, Tlr4 and Ccl21a), the focal adhesion signalling pathway (annotated with Col1a1, Itgb8, Col3a1, Mylk4, Spp1, Tnc, Col5a1, Comp, Col5a2, Fn1, Thbs4, Col1a2 and Thbs2), the protein digestion and absorption signalling pathway (annotated with Col1a1, Col14a1, Col5a1, Col1a2, Col12a1, Col3a1, Col5a2, Kcnq1 and Cpa3) and the Toll-like receptor signalling pathway (annotated with Tlr4, Ctsk, Tlr8 and Spp1) were all significantly enriched with the differentially expressed genes in the MI samples compared with the sham control samples (Table 1).

**Table 1:** KEGG pathway analysis Number* indicates the number of differentially expressed genes that are annotated in the pathway.

Term	Number*	*P* value	Genes
ECM–receptor interaction	12	5.66E−16	Col5a1, Itgb8, Col1a1, Col1a2, Fn1, Col5a2, Col3a1, Thbs4, Spp1, Tnc,Thbs2, Comp
PI3K-Akt signalling pathway	13	8.78E−10	Col5a2, Col5a1, Col3a1, Comp, Thbs4, Col1a1, Thbs2, Spp1, Itgb8, Col1a2, Fn1, Tnc, Tlr4
NF-κB signalling pathway	5	6.14E−05	Gm21541, Gm13304, Ccl21b, Tlr4, Ccl21a
TGF-β signalling pathway	3	0.006	Dcn, Tgfbr1, Tgfb3
MAPK signalling pathway	4	0.025	Tgfbr1, Ecsit, Tgfb3, Pla2g4a
Focal adhesion	13	8.94E−13	Col1a1, Itgb8, Col3a1, Mylk4, Spp1, Tnc, Col5a1, Comp, Col5a2, Fn1, Thbs4, Col1a2, Thbs2
Protein digestion and absorption	9	5.15E−11	Col1a1, Col14a1, Col5a1, Col1a2, Col12a1, Col3a1, Col5a2, Kcnq1, Cpa3
Toll-like receptor signalling pathway	4	8.59E−04	Tlr4, Ctsk, Tlr8, Spp1

### Deregulated lncRNAs are related to cardiac fibrosis

**Figure 4:**
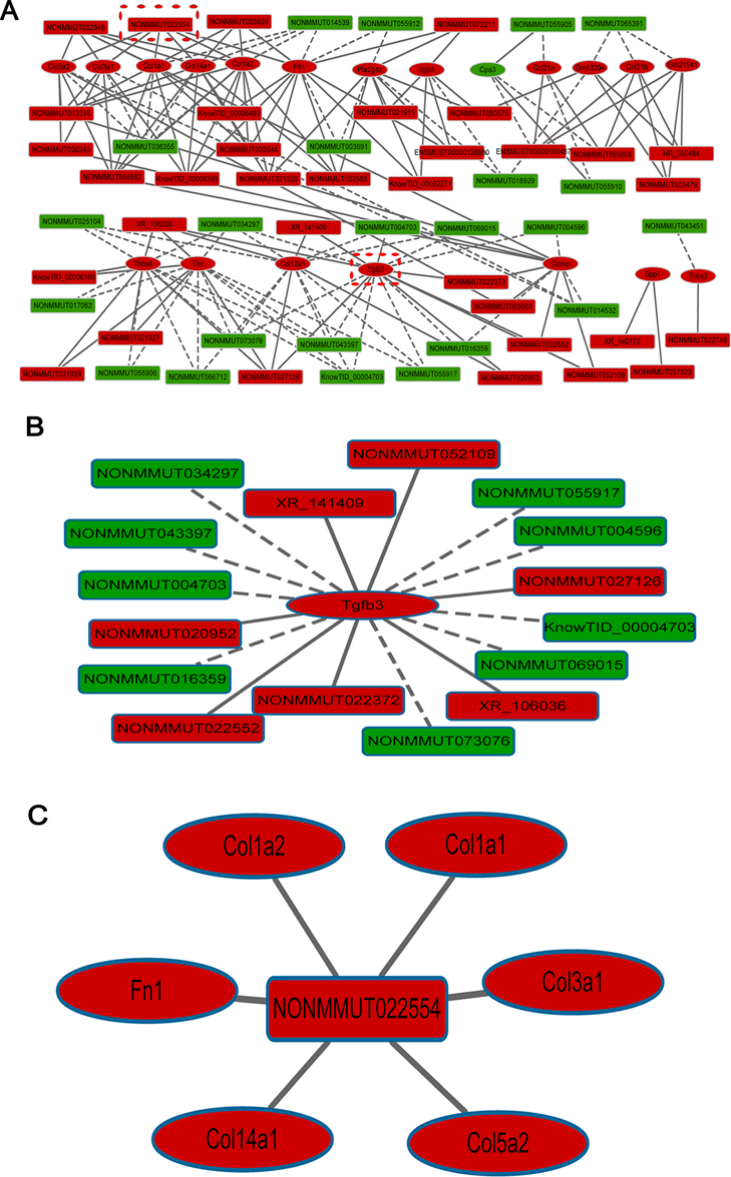
Prediction of lncRNA–mRNA association network (**A**) The co-expression network was composed of 173 connections between 57 lncRNAs and 20 coding genes. (**B**) Connections between top-ranked gene TGF-β_3_ and lncRNAs. (**C**) Connections between the top-ranked lncRNA NONMMUT022554 and coding genes. Rectangle nodes represent mRNAs and hexagon nodes represent lncRNAs. Red nodes represent up-regulated lncRNAs or mRNAs and green nodes represent down-regulated lncRNAs or mRNAs. The solid line indicates positive correlation and the dashed line negative correlation.

LncRNA and mRNA co-expression network showed that the expression of lncRNAs was significantly correlated with the expression of mRNAs that participate in the cardiac fibrosis-related pathways in Table 1. Here, our analyses were focused on the differentially expressed lncRNAs and mRNAs with fold changes more than 2.0 or less than 0.5. The network contained 173 significantly correlated lncRNA–mRNA edges (*P*<0.05) and 77 nodes, including 57 differentially expressed lncRNAs and 20 differentially expressed genes (Figure 4A and Supplementary Table S4). Among them, 34 lncRNAs were up-regulated and correlated with all 20 altered fibrosis-related coding genes and 23 down-regulated and correlated with 19 coding genes either in a positive or negative manner (Figure 4A and Supplementary Table S4). Analysis of the genomic status showed that 57 lncRNAs included 31 sense non-exonic, 19 intergenic, 5 antisense and 2 pseudogene lncRNAs (Supplementary Table S4).

In the co-expression network, the gene TGF-β_3_, a crucial component in the TGF-β signalling pathway and the MAPK signalling pathway as well, was identified as the top-ranked gene according to the number of connections with the differentially expressed lncRNAs. As illustrated in Figure 4(B), TGF-β_3_ was up-regulated in cardiac fibrosis and was negatively correlated with nine down-regulated lncRNAs (NONMMUT034297, NONMMUT043397, NONMMUT004703, NONMMUT016359, NONMMUT073076, NONMMUT069015, knowTID_00004703, NONMMUT004596 and NONMMUT055917), but positively correlated with seven up-regulated lncRNAs (XR_141409, NONMMUT020952, NONMMUT052109, NONMMUT022372, NONMMUT022552, XR_106036 and NONMMUT027126). NONMMUT022554 was the top-ranked lncRNA according to the number of connections with the differentially expressed genes. As illustrated in Figure 4(C), NONMMUT022554 was up-regulated in the cardiac fibrosis model and was positively correlated with six up-regulated genes (Col1a2, Col1a1, Col3a1, Col5a2, Col4a1 and Fn1) that are known to be involved in the ECM–receptor interaction and the PI3K-Akt signalling pathway.

We analysed the 10 up-stream and 10 down-stream neighbouring coding genes for each of the 57 differentially expressed lncRNAs and calculated the correlation for pair-wise lncRNAs and the neighbouring genes. We obtained 74 highly correlated pairs between 40 lncRNAs and their neighbouring coding genes (*P*<0.05). And 38 pairs showed positive correlation and 36 pairs showed negative correlation (Supplementary Table S5). LncRNA NONMMUT023529 showed a positive correlation with its neighbouring fibrosis-related gene Col14a1.

To identify the conservation of the 57 differentially expressed lncRNAs with humans, we mapped the candidate lncRNAs to human genome using NCBI BLASTn and detected those lncRNAs that have at least 80% identity with human, of which 9 lncRNAs were conservative to the human genome (Table 2).

**Table 2:** Conservative lncRNAs to the human genome

lncRNAs	Chromosome	Fold change
NONMMUT032848	chr18	2.282396
NONMMUT027126	chr16	2.841048
NONMMUT032844	chr18	2.419761
NONMMUT021915	chr14	2.046211
NONMMUT003479	chr1	2.994935
NONMMUT072211	chrX	2.089519
NONMMUT030245	chr17	2.052835
NONMMUT073076	chrX	−2.283268
NONMMUT069015	chr9	−3.399379

We used the VISTA Enhancer Browser (http://enhancer.lbl.gov/) a central resource for experimentally validated human and mouse non-coding fragments with gene enhancer activity to analyse the changed lncRNAs. Among the 57 differentially expressed lncRNAs, no enhancer-like lncRNAs were detected.

## DISCUSSION

Cardiac gene profiling studies have had enormous impacts on our understanding of the cardiac pathology, pointing to the biological heterogeneity of specific disorders, providing identification of novel regulators of cardiac gene expression and function and defining the pathways that interplay to promote cardiovascular diseases. In the present study we acquired a number of evidence for the possible involvement of lncRNAs in cardiac fibrosis. First, approximately 1% of lncRNAs tested (545 of the 55000) and mRNAs (209 of 23000) were found significantly deregulated in their expression in peri-infarct tissue with cardiac fibrosis in mice compared with the sham control group. Second, GO and pathway analyses revealed that the deregulated genes primarily include those known to be related to the development of cardiac fibrosis. Finally, 173 matched lncRNA–mRNA pairs were established for 57 differentially expressed lncRNAs and 20 differentially expressed coding genes. These findings suggest the role of lncRNAs in the development of cardiac fibrosis.

As reported by previous studies, MI can result in left ventricular dilatation associated with interstitial fibrosis, fibroblasts proliferation and heart remodelling [7,30]. An important part of the MI remodelling process is formation of fibrotic tissue which contributes to the development of cardiac contractive dysfunction and arrhythmias. Multiple factors and signalling pathways, including gene products and non-coding RNAs, have been shown to be involved in cardiac fibrosis, either directly or indirectly. It has been reported that lncRNAs regulate senescence process and viability of fibroblasts [10] and are associated to heart failure and maladaptive remodelling [11]. An *in vitro* study showed the deregulated lncRNAs in adult rat cardiac fibroblasts after AngII treatment [29]. Recently, Ounzain et al. [31] systematically analysed and characterized lncRNAs in a mouse model of MI. They identified 1521 novel unannotated lncRNAs and hundreds of novel heart-specific lncRNAs which are correlated with unique regulatory and functional characteristics relevant to maladaptive remodelling, cardiac function and cardiac development. Some novel lncRNAs are dynamically regulated in response to MI. The study also revealed that the enhancer-associated lncRNAs are more enriched in the nucleus, whereas the lncRNAs involved in post-transcriptional and translational processes are mainly cytoplasmically located. Importantly, they found hundreds of human orthologues of novel lncRNAs, some of them are regulated in human cardiac disease [31].

Here we demonstrated for the first time in an *in vivo* model of cardiac fibrosis that lncRNAs have the potential to regulate cardiac fibrosis. Among the 57 differentially expressed lncRNAs, 34 lncRNAs were up-regulated and correlated with all of the 20 fibrosis-related coding genes and 23 were down-regulated lncRNAs and correlated with 19 genes either in a positive or negative manner, indicating that up- and down-regulated lncRNAs may be involved in the balanced regulation of fibrosis. For instance, nine down-regulated and seven up-regulated lncRNAs were negatively and positively correlated with TGF-β_3_ up-regulation respectively. Up-regulated NONMMUT022554 was positively correlated with six up-regulated genes that are involved in the ECM–receptor interaction and the PI3K-Akt signalling pathway which participate in the process of cardiac fibrosis. In addition, 40 lncRNAs show positive or negative correlation with their neighbouring genes; 9 lncRNAs are conservative to the human genome, indicating their potential role in the human heart.

We performed GO and pathway analyses to forecast the biological functions of target genes and the potential roles of the differentially expressed lncRNAs in cardiac fibrosis. We found that the deregulated transcripts were mostly enriched in GO terms of heparin binding, calcium binding, ECM structural constituent and collagen binding. Furthermore, we found that the altered transcripts were primarily associated with eight pathways: ECM–receptor interaction, PI3K-Akt, nuclear factor κB (NF-κB), TGF-β, MAPK, the focal adhesion, protein digestion and absorption and Toll-like receptor signalling pathways. We also characterized the top-ranked lncRNAs in the lncRNA–mRNA co-expression network based on their possible connections to cardiac fibrosis-relevant protein-coding genes. We identified TGF-β_3_ as the top-ranked gene, which plays a critical role in the TGF-β signalling pathway and MAPK signalling pathway. These two pathways were reported to take part in the cardiac fibrosis. Further studies of these lncRNAs should provide useful insight into their actual roles in cardiac fibrosis. NONMMUT022554 was the top-ranked lncRNA that was positively correlated with six up-regulated genes known to be involved in ECM–receptor interaction and the PI3K-Akt signalling pathway in the pathway analysis.

Differential expression of lncRNAs in relation to fibrotic processes has been reported in various organs/tissues, such as pulmonary, renal, peritoneal and cystic fibrosis [32–36], but has not been documented in cardiac fibrosis. In order to identify the possible overlaps of the deregulated lncRNAs between cardiac fibrosis and other tissues mentioned above, we used chromosomal location of lncRNAs to search for common lncRNAs between the 57 fibrosis-associated lncRNAs identified in our study and deregulated lncRNAs in other studies [32–36]. However, we failed to find any common lncRNAs between our study and others. This could be explained by the low conservation of lncRNAs across tissues and species, different pathological processes produced by different causes/inducers and dynamic lncRNAs expression. Indeed, similar discrepancy was noticed by Sun et al. who reported that there is limited overlap between the changed lncRNAs in paraquat-induced lung fibrosis in mouse [32] and bleomycin-induced lung fibrosis in rat [33].

LncRNAs may be involved in the regulation of fibrogenic process through various mechanisms [32–35]. The MAPK pathway is targeted by the altered lncRNAs in lung fibrosis [32] and peritoneal fibrosis in mice [35]; the TGF-β pathway is closely associated with the changed lncRNAs in kidney fibrosis [34] and peritoneal fibrosis in mice [35]; the Jak/STAT signalling pathway is associated with the deregulated lncRNAs in lung fibrosis in rats [33] and mouse peritoneal fibrosis [35]; the TGF-β and MAPK signalling pathways are related to the changed lncRNAs characterized in our study. It can therefore be concluded that although expression profiles of lncRNAs lack general conservations among different fibrotic tissues, they may regulate fibrotic processes through conserved mechanisms.

We found that the deregulated lncRNAs and coding genes are involved in eight pathways associated with the process of cardiac fibrosis in mice. Most of the deregulated genes identified in our study have known biological functions. For instance, KCNQ1 encodes slow delayed rectifier K^+^ current (*I*_Ks_) that is a major component of cardiac membrane repolarization and plays a critical role in determining action potential duration [37]. TLRs 2 and 8 genes are related to activation of monocytes to release pro-inflammatory cytokines [38]. TGFBR1 is essential for the remodelling process of the cushions and promotes mesenchymal cell differentiation to vascular smooth muscles in heart [39]. Tnc is an important component of the regulatory thin filament complex that coordinates muscle contraction [40].

It must be noted that the peri-infarct tissues used for expression profiling in our study contain not only fibrotic tissues, but also other types of cells including cardiomyocytes, endothelial cells and smooth muscle cells. Thus the observed changes of expression of lncRNAs and mRNAs cannot be ascribed solely to cardiac fibrosis. Cardiomyocyte apoptosis [41] and angiogenesis [42] are also involved in myocardial remodelling after MI. However, the present study only focuses on analysis of the potential roles of lncRNAs in cardiac fibrosis. Other possible roles of these altered lncRNAs will be investigated in future studies.

In conclusion, we have identified for the first time the lncRNA expression signature in peri-infarct region containing significant cardiac fibrosis induced by MI in mice. We have also characterized the possible involvement of lncRNAs in cardiac fibrosis by bioinformatics analyses. However, detailed studies are needed to explore the actual functional significance of the deregulated lncRNAs in cardiac fibrosis.

## Abbreviations

AngII: angiotensin II
ECM: extracellular matrix
EF: ejection fraction
FDR: false discovery rate
FS: fractional shortening
KEGG: Kyoto Encyclopedia of Genes and Genomes
lncRNA: long non-coding RNA
MAPK: mitogen activated protein kinase
MI: myocardial infarction
NF-κB: nuclear factor κB
PI3K-Akt: phosphoinositid-3 kinase/protein kinase B
qRT-PCR: quantitative real-time PCR
TGF-β: transforming growth factor-β
